# Using DNA Origami to Study Nanoscale Organization of Plasma Membranes

**DOI:** 10.1021/acs.nanolett.6c00255

**Published:** 2026-04-20

**Authors:** Eloina Corradi, Konlin Shen, Zeynep Karatas, Maureen Cercy, Thomas Schlichthaerle, Margaux Caumont, Mélissande Osouf, Brune Vialet, Philippe Barthelemy, Morgane Rosendale, Adiyodi Veetil Radhakrishnan, Tianchi Chen, Ralf Jungmann, Arnaud Gissot, Shawn M. Douglas, Grégory Giannone

**Affiliations:** † University Bordeaux, CNRS, 131738 IINS, UMR 5297, Bordeaux, F-33000, France; ‡ Dept. of Cellular and Molecular Pharmacology, 8785 University of California San Francisco, San Francisco, California 94158, United States; § Faculty of Physics and Center for Nanoscience, 9183 LMU Munich, Munich, 80539, Germany; ∥ Research Group Molecular Imaging and Bionanotechnology, Max Planck Institute of Biochemistry, Martinsried, 82152, Germany; ⊥ ARNA, INSERM U1212, CNRS 5320, Université de Bordeaux, Bordeaux, F-33076, France; # Somaiya Centre for Integrated Science Education and Research (SciSER®), Somaiya Vidyavihar University, Mumbai, Maharashtra 400077, India

**Keywords:** DNA origami, plasma membrane nanodomains, single particle tracking, mechanobiology, actin cytoskeleton

## Abstract

Plasma membrane (PM) lipids and proteins partition into nanodomains that regulate cellular processes by controlling local membrane organization. However, nanodomains’ small size and temporal instability hinder their study in living cells. To address this, we built fluorescent DNA origami probes that insert into the PM via lipid anchors displayed on cells. Using DNA origami allows precise control over anchor number and spatial arrangement, enabling nanometer-scale sampling of the PM. Once inserted, probes diffusing across the membrane are followed by single-particle tracking to survey the PM landscape. Varying lipid anchor number and arrangement shows that origami immobilization requires simultaneous interactions with multiple nanodomains. Disrupting the actin cytoskeleton reduced immobilization, confirming its role in nanodomain stability. Moreover, acute cell stretching transiently increases origami mobility, indicating that mechanical forces can reversibly regulate PM nanodomain organization. This novel membrane-integrated DNA origami approach provides mechanistic insights into PM nanodomain architecture and dynamics in living cells.

## Graphical abstract

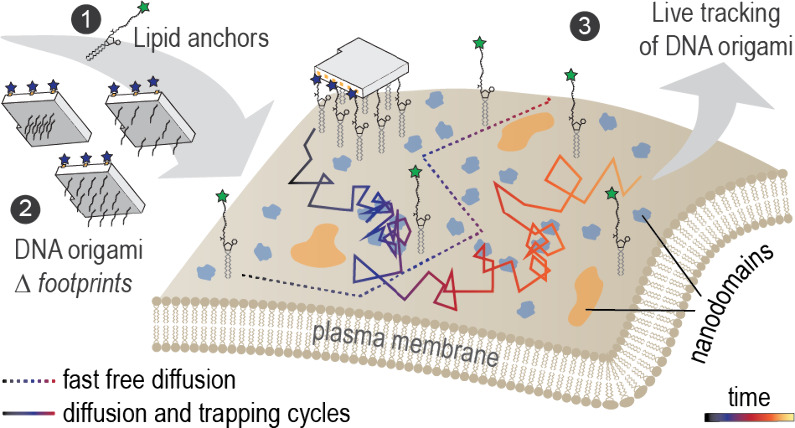

The plasma membrane (PM) is a central platform for signaling, mechanotransduction, and cytoskeletal organization.^1,2^ Its function depends on the lateral heterogeneity of its lipid and protein components that generate functional domains.^3^ While some domains extend over micrometers, they are fundamentally built from nanoscale structures (10–200 nm), called nanodomains, that compartmentalize and coordinate membrane-based activities.^4,5^ PM nanodomains regulate key signaling pathways involving Ras small GTPases,^6^ Src-family kinases^7^ and interferon-γ receptor (IFN-γR),^8^ and contribute to several cellular processes such as immune response to cancer,^9^ cell adhesions^10^ and migration,^11^ phagocytosis,^12^ and mechanotransduction.^11,13^ These functions are often mediated by transmembrane proteins such as integrins^10,11^ and CD44 ^12,14^ that link the extracellular matrix (ECM) to the actin cytoskeleton. Moreover, PM nanodomains also organize membrane topography, enabling processes such as cargo endocytosis^15^ and viral budding.^16^ Despite their importance, the dynamic nanoscale organization of PM nanodomains remains poorly understood.

Our understanding of how PM nanodomains coordinate cellular processes has been limited by the challenges of visualizing these small, dynamic structures. Current knowledge primarily relies on advanced optical techniques such as super-resolution microscopy and single-particle tracking (SPT), as conventional microscopy lacks the spatiotemporal resolution required to study PM nanodomains.^5^ Super-resolution imaging of fixed samples offers high spatial resolution and wide sampling areas, but cannot capture dynamic behavior.^13,17^ Near-field single-molecule optical microscopy (NSOM)^10^ and homo-FRET^18^ achieve nanometer-scale precision (∼5 nm), but suffer from low temporal resolution (seconds) and, for NSOM, limited fields of view. In contrast, SPT-based methods offer milliseconds temporal resolution with localization precision of 2–30 nm. However, this comes at the cost of reduced sampling area (e.g., STED-FCS^19,20^ ) and limited molecular coverage, as they typically track individual proteins (e.g., GPI-anchored proteins)^7,21,22^ or lipids (cholesterol, sphingolipids),^19,20,23–25^ providing only partial insight into overall PM nanodomain organization.

DNA origami enables the folding of a long single-stranded DNA (ssDNA) scaffold into a user-defined structure with single-base precision, resulting in a nanoparticle that can be sculpted with single-nanometer resolution.^26^ Since the introduction of the technique in 2006, DNA origami has evolved from simple planar 2D architectures into complex microscale 3D assemblies, with burgeoning applications in materials science, photonics, and biology.^27,28^ Importantly, the high resolution addressability of DNA origami has cemented DNA origami as a powerful platform to control ligand–receptor mechanisms by spatially organizing either receptors^29,30^ or ligands.^31–33^ DNA nanostructures patterned with lipophilic anchors have been used to interface with and shape both model^34–38^ and cellular membranes,^39,40^ to induce deformation,^35,38^ curvature,^36^ perforation^38,40^ or offer protection.^39^ However, despite their nanometric precision and versatility, DNA origami have not yet been applied to investigate PM nanodomain organization and dynamics in living cells. Here, building on our previous DNA origami pegboards,^29,30^ we developed a DNA-origami-based approach to probe PM nanodomains in living cells with both high temporal resolution (millisecond range) and spatial localization precision (∼30 nm), while simultaneously sampling larger areas (38.5 nm × 36 nm) of the PM. We engineered a DNA origami chassis to interrogate the organization of PM nanodomains through hybridization with complementary ssDNA-conjugated lipid anchors inserted into the PM. The precise spatial arrangement of our handles enables interrogation of membrane features smaller than 30 nm, far lower than the optical resolution limit. We decorated the origami chassis with multiple fluorophores to ensure a longer-lasting fluorescent signal without compromising the field of view. By embedding these DNA origami probes into cell PMs and measuring their diffusion characteristics using single particle tracking,^41–44^ we provide new insights into PM nanodomain organization and their response to global perturbations such as actin destabilization and cell mechanical forces.

## DNA Origami Tracking Can Be Used to Probe Cell Membrane

Our PM nanodomain probes are based on a “molecular pegboard” design previously used to probe T cell activation^30^ and phagocytosis.^29^ We folded an 8064-nt ssDNA scaffold into a roughly 60 nm x 60 nm x 6 nm rectangular prism. On one side of the structure, we added 12 biotin sites for conjugating monomeric-Streptavidin labeled with ATTO594 fluorophore (mStravATTO594). On the other side of the structure, we designed 72 uniquely addressable sites where a 16-nt ssDNA handle for probing the membrane could be extended. The sites were separated from each other by 3.5 nm in one direction and 7.2 nm in the other direction, allowing for PM interrogation at the nanometer scale (Figure 1a, Figure 1b).

**Figure 1:**
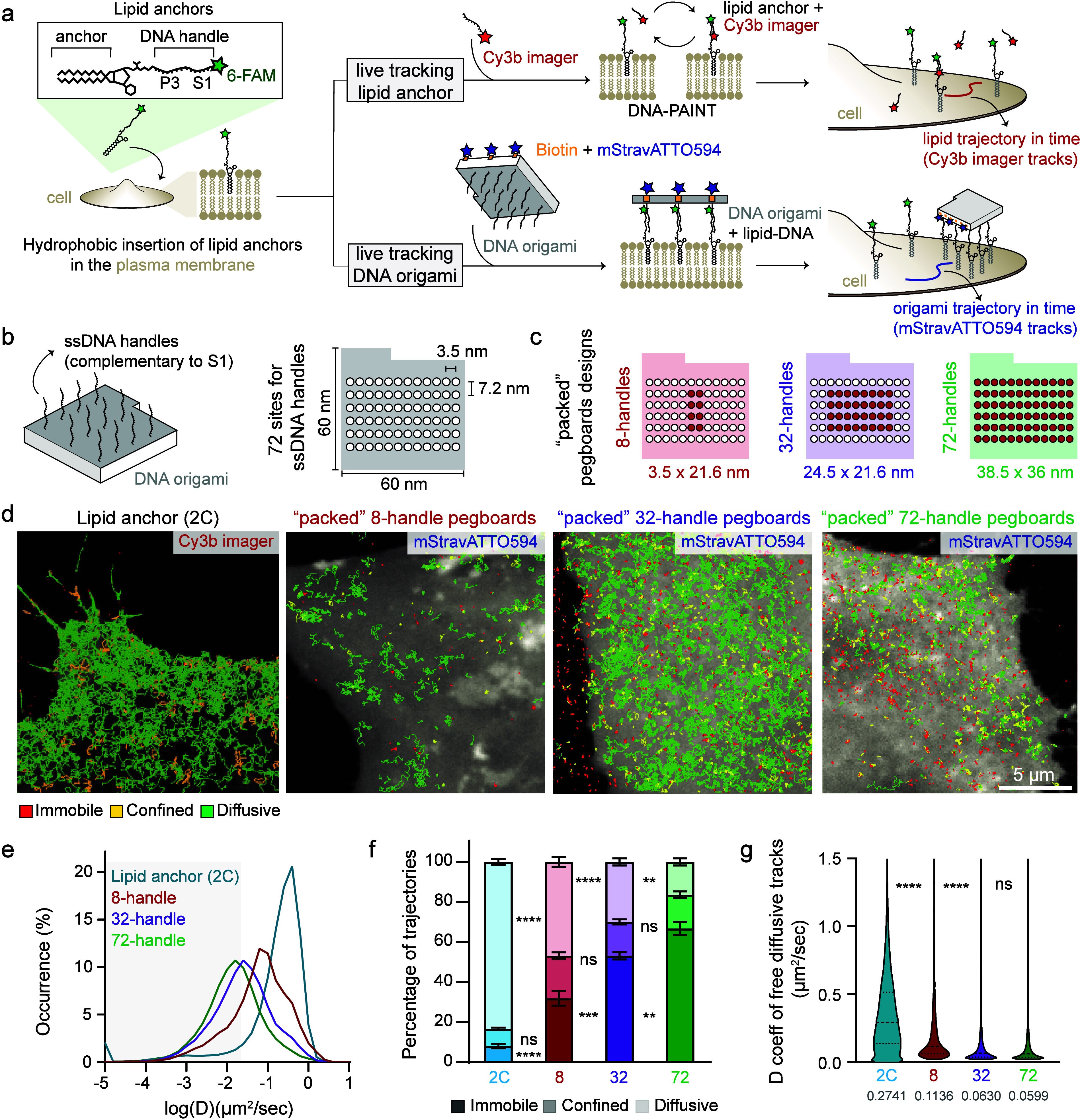
**DNA origami diffusion in the PM depends on the number of handles for lipid anchors. a)** Schematic of the experimental strategy: DNA origami displaying arrangements of ssDNA handles are hybridized to synthetic lipids anchors displayed on cell membranes. Using single particle tracking, we measure the diffusion characteristics of the DNA origami across the cell surface to probe the nanoscale landscape of the plasma membrane in living cells. Single lipids are tracked through hybridization with a P3 Cy3b imager. **b,c)** Schematic of the DNA origami pegboard (b) and of the three “packed” designs with 8, 32, or 72 ssDNA handles for lipid anchors (c). **d)** Trajectories of lipid anchors or DNA origami overlaid on 6-FAM cell plasma membrane (gray). Trajectories are color coded to show their diffusion modes: diffusive (green), confined (yellow) and immobile (red). **e)** Distributions of the mean diffusion coefficient D computed from the trajectories of lipid anchors 2C or DNA origami. **f)** Fractions of tracked lipid anchors 2C and DNA origami undergoing free diffusion, confined diffusion, or immobilization in the plasma membrane. **g)** Diffusion coefficient D for all free diffusive tracks of the lipid anchors 2C and DNA origami. Median values reported. Data information: mean ± SEM (f), median and interquartile range (g). Statistics: n = 5 (2C), n = 6 (8-handles), n = 7 (32-handles), n = 6 (72-handles), e) 2-way ANOVA with Tukey multiple comparisons test, f) number of diffusive tracks 132543 (2C), 7312 (8-handles), 8487 (32-handles), 14864 (72-handles), Kruskal–Wallis test with Dunn’s multiple comparisons test. Abbreviations: 2C, lipid anchor with two aliphatic chains.

To enable interactions between the cells and the pegboards, we decorated the cells with lipid anchors conjugated to ssDNA strands (Figure 1a, see Method for details). The first 11 bases on the 5′ end of the lipid-anchor strand hybridize to an “imager” strand, carrying a Cy3b fluorophore (P3 site, Figure 1a, Figure S1a). This 11-base sequence was designed for transient binding to allow live tracking of the lipid anchors via DNA-PAINT (Figure 1a). The last 16 bases of the lipid-anchor ssDNA strands are complementary to the ssDNA handles displayed on the DNA origami pegboard (S1 site, Figure 1a, ). The length and sequence of the handles were chosen to minimize secondary structure formation as well as to stabilize duplex formation at room temperature.

Because DNA origami diffusion on lipid membranes depends on lipid composition,^45^ we synthesized two different lipid anchors bearing long saturated aliphatic chains, characteristic of PM nanodomains,^46^ a single 18-carbon anchor (1C) and a double-chain 15-carbon anchor (2C) (). Lipid anchors were conjugated to 6-carboxyfluorescein (6-FAM) at the 3′ end of ssDNA to monitor PM incorporation in cultured mouse embryonic fibroblasts (MEFs) (). Fluorescence imaging showed that both 1C and 2C lipid anchors were incorporated into the outer leaflet through hydrophobic insertion (). Addition of Cy3 imager strands enabled tracking of thousands of trajectories for both lipid anchors (, Movie S1). Control experiments without lipid anchors showed no detectable tracks, confirming trajectory specificity. For trajectories exceeding 200 ms (>10 points), we computed the mean squared displacement (MSD) and diffusion coefficients (D) (). By fitting the MSD over time, we classified diffusion modes of lipid anchors as immobile, confined, or free diffusive^41^ (). MSD analysis revealed that both types of lipid anchors predominantly diffuse freely within the membrane, with minimal confined or immobile fractions (). The 2C lipid anchor exhibited higher diffusion coefficients than 1C anchor, indicating faster free diffusion (). Validation experiments at 500 Hz acquisition confirmed free diffusion dominance and the faster 2C versus 1C dynamics (). This might be counterintuitive, as a 2C anchor would be expected to generate greater membrane frictional drag; however, its faster diffusion may arise from differences in membrane insertion and molecular geometry.^46–48^ Single-chain anchors tend to adopt a cone-shaped geometry that perturbs lipid packing, whereas two-chain anchors form a more cylindrical, lipid-like structure. In addition, the 18-carbon chain of the 1C anchor may insert deeper into the PM than the 15-carbon chains of the 2C anchor, increasing lipid interactions or inducing local membrane deformation that could reduce lateral mobility. Nonetheless, both anchors show fast free diffusion as dominant mode of motion, indicating that individual synthetic lipids behave similarly to native saturated lipids,^19,20,25^ and providing a foundation for DNA origami-based nanodomain organization studies.

We next confirmed that DNA origami pegboards diffuse on the PM when bound to lipid anchors. Using a test DNA origami structure that had 12 DNA handles, we tracked pegboards during live imaging. Without lipid-anchor insertion in the PM, no origami tracks were detectable on cells. However, once we decorated the cells with lipid anchors, the DNA origami attached to the cell membranes and could be tracked (). Compared to individual lipids, the DNA origami particles exhibited decreased free diffusion and increased immobilization, regardless of having 1C or 2C lipid anchors (). Unlike individual lipids, the DNA origami did not exhibit significantly different diffusion coefficients or fraction of diffusion modes when bound to 1C or 2C lipid anchors (), likely due to dominant viscous drag effects in the membrane. We selected 2C lipid anchors for subsequent experiments, matching the dual aliphatic chain structure typical of PM lipids. These results demonstrate that, while individual lipids remain largely free-diffusing, DNA origami bound to multiple lipid anchors display confined diffusion and immobilization on the PM, thus confirming the feasibility of the proposed strategy to probe nanoscale organization of the PM.

## Pegboard Diffusion in the PM Depends on the Number of Handles for Lipid Anchors

The increased immobilization and slower diffusion of DNA origami pegboards compared to single lipids could arise from interactions with lipid anchors within PM nanodomains, interactions with native extracellular PM components, unintended pegboard aggregation, or a combination of all three. To distinguish among these possibilities, we varied the number of handles per pegboard (Figure 1c). If immobilization were dominated by pegboard–pegboard or pegboard–extracellular interactions, diffusion behavior would be largely independent of handle number. Conversely, a dependence on handle number would indicate that increased membrane engagement enhances interactions with PM nanodomains.

We generated three pegboard variants with either 8, 32, or 72 handles, designed to bind lipid anchors across 3.5 nm × 21.6 nm, 24.5 nm × 21.6 and 38.5 nm × 36 nm areas, respectively (Figure 1c). After incorporating 2C lipid anchors into MEF cell membranes, we added pegboards and quantified their diffusive behavior using single particle tracking (SPT) (Figure 1d). Higher handle numbers correlated with increased immobilization: 8-handle pegboards showed the highest diffusive trajectory percentage, followed by 32-handle and 72-handle pegboards (Figure 1d-f). Diffusion coefficients decreased with handle number (Figure 1e,g). These results demonstrate that pegboard diffusion on the PM is dictated by lipid anchor–membrane interactions rather than on the DNA origami pegboard itself, validating the relevance of our DNA origami particles as a tool for probing the organization of PM nanodomains.

## Spatial Arrangement of Lipid Anchors Influences DNA Origami Diffusion

We wondered if the observed changes in the pegboard’s diffusive properties stem solely from increased membrane drag triggered by a higher number of handle-lipid anchors. To determine whether we were probing PM organization or simply seeing the effect of more points of contact between the origami and the cell PM, we limited the pegboard handle number to 8, but varied the spatial arrangement of the handles to change the membrane footprint of the pegboards, alongside a 72-handle control (Figure 2a,b). After inserting lipid anchors into cell membranes and mixing with pegboards, we tracked pegboard diffusion along the cell membrane. All three DNA origami configurations with 8 handles (“packed”, “patchy”, or “spread”) showed a strong decrease in the fraction of immobile tracks compared to the 72-handles control (Figure 2c,d, Movie S2, Movie S3, Movie S4). Notably, the 8-handle ″packed″ and ″patchy″ designs showed similar diffusion properties, while the ″spread″ design exhibited more immobile trajectories (Figure 2c-e). Diffusion coefficient analysis of freely diffusive trajectories revealed that the ″packed″ design diffused fastest, followed by ″patchy,″ then ″spread″ and 72-handle designs (Figure 2d,f). This order corresponds directly to the PM area bounded by the DNA origami handles: ″packed″ designs covered the smallest area, followed by ″patchy,″ and then the ″spread″ and 72-handle designs covering equivalent areas (Figure 1c, Figure 2b). These results demonstrate that pegboard–PM interactions are governed by the spatial arrangement of lipid anchors rather than their absolute number. Since individual lipid anchors diffuse freely in the membrane, reduced pegboard diffusion reflects the intrinsic PM spatial organization, validating our approach for probing PM nanodomains.

**Figure 2:**
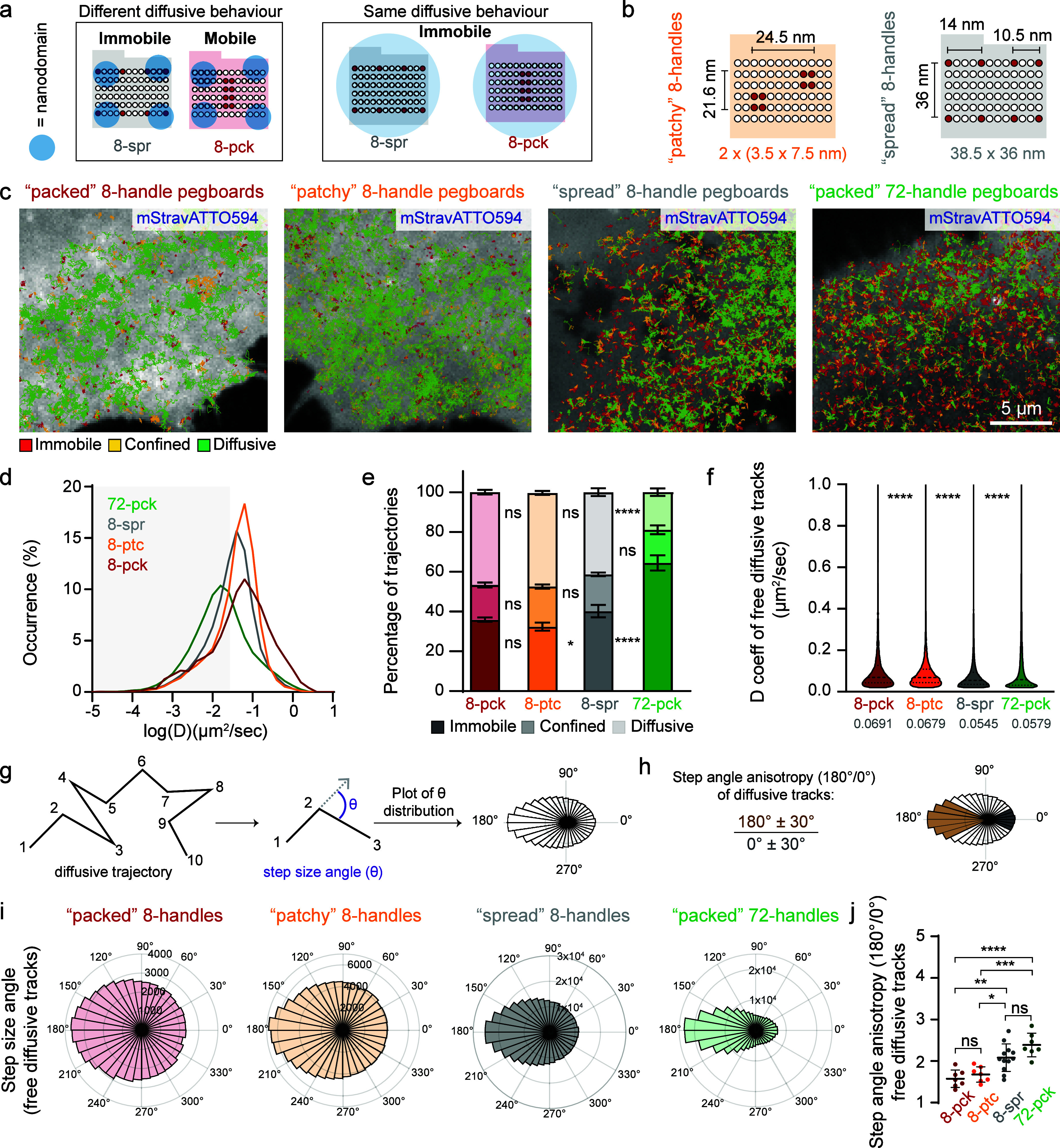
**Spatial arrangement of lipid anchors influences DNA origami diffusion and anisotropy. a,b)** Schematic of the tested hypothesis (a) and DNA origami designs (b). All origami displayed 8 handles for lipid anchors but varied in their handle arrangement. **c)** DNA origami trajectories overlaid on 6-FAM cell plasma membrane (gray). Trajectories are color coded to show their diffusion modes: diffusive (green), confined (yellow) and immobile (red). **d)** Distributions of the mean diffusion coefficient D computed from DNA origami trajectories. **e)** Fractions of tracked DNA origami undergoing free diffusion, confined diffusion or immobilization in the plasma membrane. **f)** Diffusion coefficient D for all free diffusive tracks of the DNA origami. **g,h)** Schematic of the step size angle and anisotropy analysis. **i)** Representative step size angle distribution for the diffusive tracks of the tested DNA origami. **j)** Step angle anisotropy of all the diffusive tracks. Data information: mean ± SEM (e,j), median and interquartile range (f). Statistics: *n* = 5 (8-pck), *n* = 7 (8-ptc), *n* = 12 (8-spr), *n* = 6 (72-pck), d) 2-way ANOVA with Tukey multiple comparisons test, f) number of diffusive tracks 13648 (8-pck), 26862 (8-ptc), 75901 (8-spr), 23228 (72-pck), Kruskal–Wallis test (nonparametric one-way ANOVA) with Dunn’s multiple comparisons test. j) ordinary one-way ANOVA Tukey multiple comparisons test, each dot corresponds to the step angle anisotropy per single cell. Abbreviations: pck, packed; ptc, patchy; spr, spread.

## Spatial Arrangement of Lipid Anchors Affects the Anisotropy of DNA Origami Diffusion

To further confirm that DNA origami pegboard diffusive properties reflect PM nanodomain interactions, we analyzed trajectory isotropy for free-diffusing and confined trajectories. This analysis captures transient confinement events that may be missed in global diffusion classification, where trajectories that appear to be overall free-diffusive could contain brief confined periods. To assess trajectory isotropy, we measured step-size angles for every diffusive trajectory and plotted distributions for each pegboard type (Figure 2g). We quantified anisotropy using ″fold anisotropy″, meaning the ratio of steps with ∼ 180° reorientations to steps with ∼ 0° reorientations (straight travel) (Figure 2h).^49^ Higher anisotropy indicates greater confinement, as confined particles experience more frequent large reorientations and fewer forward steps, while more isotropic trajectories indicate free diffusion. The ″packed″ and ″patchy″ 8-handle pegboards exhibited broad step-size angle distributions and low fold anisotropy values (Figure 2i,j). Conversely, ″spread″ 8-handle and 72-handle pegboards showed narrower angle distributions and significantly higher fold anisotropy (Figure 2i,j). This increase in anisotropy correlates directly with the increased PM area bounded by the different designs. Our analyses of angles from confined DNA origami tracks revealed similar trends across all four designs (), with significant differences between ″packed″ and ″spread″ 8-handle variants. These findings indicate that the plasma membrane contains a high density of nanodomains. Their size is inferred to be below ∼ 20 nm from the geometry-dependent diffusive behavior of DNA origami platforms with defined footprints. This indirect measure circumvents the ∼ 33 nm pointing accuracy of conventional single-particle tracking, which precludes their direct optical resolution. Compact platforms navigate freely through the membrane, whereas designs spanning 38.5 nm × 36 nm are effectively trapped (Figure 2a). The differential immobilization, diffusion coefficients, and anisotropy suggest that, while compact platforms primarily interact with individual nanodomains, larger platforms span multiple proximal nanodomains simultaneously, resulting in enhanced confinement (Figure 2a, ).

## DNA Origami Tracks Display Diffusion-Confinement Cycles

To gain deeper insight into the organization of PM nanodomains, we extended our analyses beyond global diffusion behavior. Reasoning that DNA origami local confinements (i.e., immobilization and confined diffusion) reflect nanodomain organization, we focused specifically on characterizing these regions. To this end, we selected the 72-handle DNA origami design, which showed the greatest degree of confinement and the highest likelihood of engaging multiple nanodomains simultaneously. We performed subtrack analysis on long-duration trajectories (>50 frames) to resolve how confinement evolves in both space and time. This approach allowed us to identify local confined subregions within single trajectories, interspersed with free-diffusive periods, and to quantify changes in diffusivity along the path of individual probes (Figure 3a,b; Movie S5, see Methods for details). From the MSD analysis of each subtrack, we first computed the radius of confinement (R_conf_), which provides an estimate of the spatial extent over which the probe remains locally constrained. The R_conf_ revealed pronounced local confinement, with a mean radius of 48.02 ± 0.58 nm and with 33.47% of subtracks exhibiting confinement radii below the system’s localization precision (pointing accuracy, σ = 33 nm, Figure 3c), and only 3.34% having radii above 100 nm. Notably, the time spent in confined states significantly exceeded time spent in free-diffusing states (Figure 3d). Together, these findings support a model in which individual DNA origami transiently engage multiple discrete, high-density nanodomain areas, giving rise to complex, dynamic confinement behavior. These dynamics likely arise from multiple sources of nanoscale membrane heterogeneity, including lipid composition and packing, lipid–protein organization, membrane nanotopography and cytoskeleton-mediated compartmentalization.

**Figure 3:**
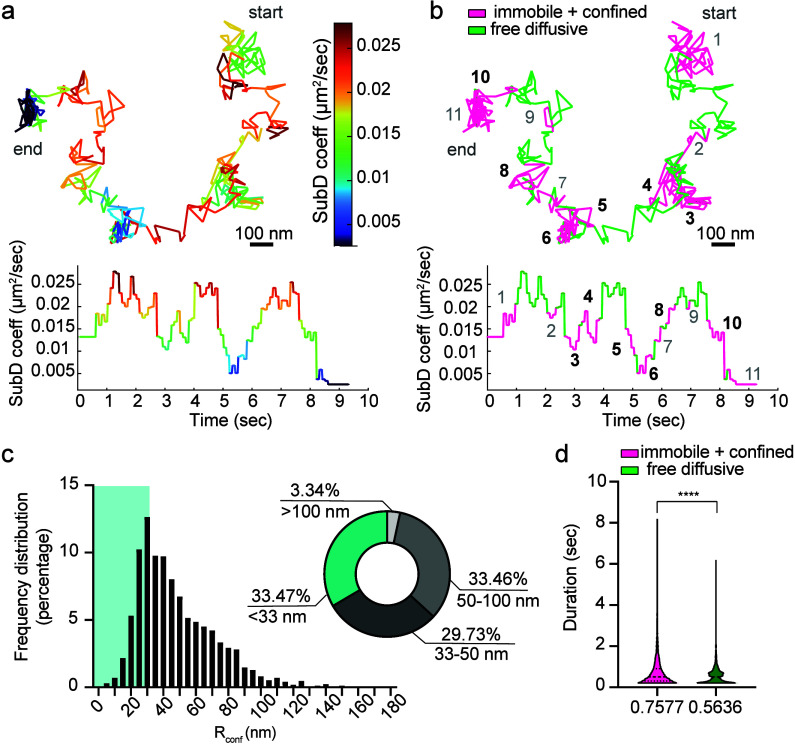
**DNA origami tracks display diffusion-confinement cycles. a)** Subtrack diffusion coefficient, D, as a function of time and space for a representative trajectory from a freely diffusing origami. **b)** Single trajectory and subtrack diffusion coefficient plot, color coded to show the subdiffusion modes: free diffusion (green), confined diffusion and immobilization (magenta). Bolded numbers correspond to regions passing the selection criteria: the subtrack must be embedded between free diffusive subtracks and has a minimum length of 10 frames. **c)** Histogram and pie plot showing the distribution of confinement radii for origami undergoing confined diffusion or immobilization. The light green shaded region marks values below the localization precision (σ = 33 nm). **d)** Duration in seconds of the different subdiffusion modes: free diffusion (green), confined diffusion and immobilization (magenta). Mean values are reported. Data information: median and interquartile range (d). Statistics: n = 1450 (number of total trajectories longer than 50 frames), number of confined and immobile regions 4120 (magenta), and of free diffusing regions 2801 (green), n = 1709 (R_conf_ values). Unpaired Mann–Whitney *t* test (d). Abbreviations: SubD, diffusion coefficient D computed on subtracks; R_conf_, radius of confinement.

## Membrane Nanodomain Stability Partially Relies on Actin

PM nanodomains interact indirectly with the actin cytoskeleton and these interactions are crucial for forming and stabilizing nanodomains.^11,50,51^ Consequently, PM nanodomains are sensitive to the underlying cortical actin network^8,12,52^ with actin filament destabilization by Latrunculin treatment inducing decreased sphingomyelin lipid entrapment^52^ and reduced PM nanodomain size.^17^ Thus, we hypothesized that if our pegboards were interacting with PM nanodomains, actin cytoskeleton disruption should affect their diffusion. We performed live tracking of 72-handle pegboards on lipid anchor-decorated cells treated with and without LatrunculinA (LatA). Actin depolymerization mediated by LatA decreased the fraction of immobile 72-handle platforms by 23% (Figure 4a-c). Further analysis revealed that 72-handle pegboards on LatA-treated cells exhibited faster free diffusion than those on untreated cells (Figure 4d), while we observed no differences in fold anisotropy for free diffusive and confined tracks (). These results demonstrate that the actin cytoskeleton significantly influences pegboard diffusion, and together with the preceding findings, they support a model in which DNA origami probe an integrated PM landscape shaped by multiple structural and organizational factors, including high-density nanodomains and cytoskeletal organization.

**Figure 4:**
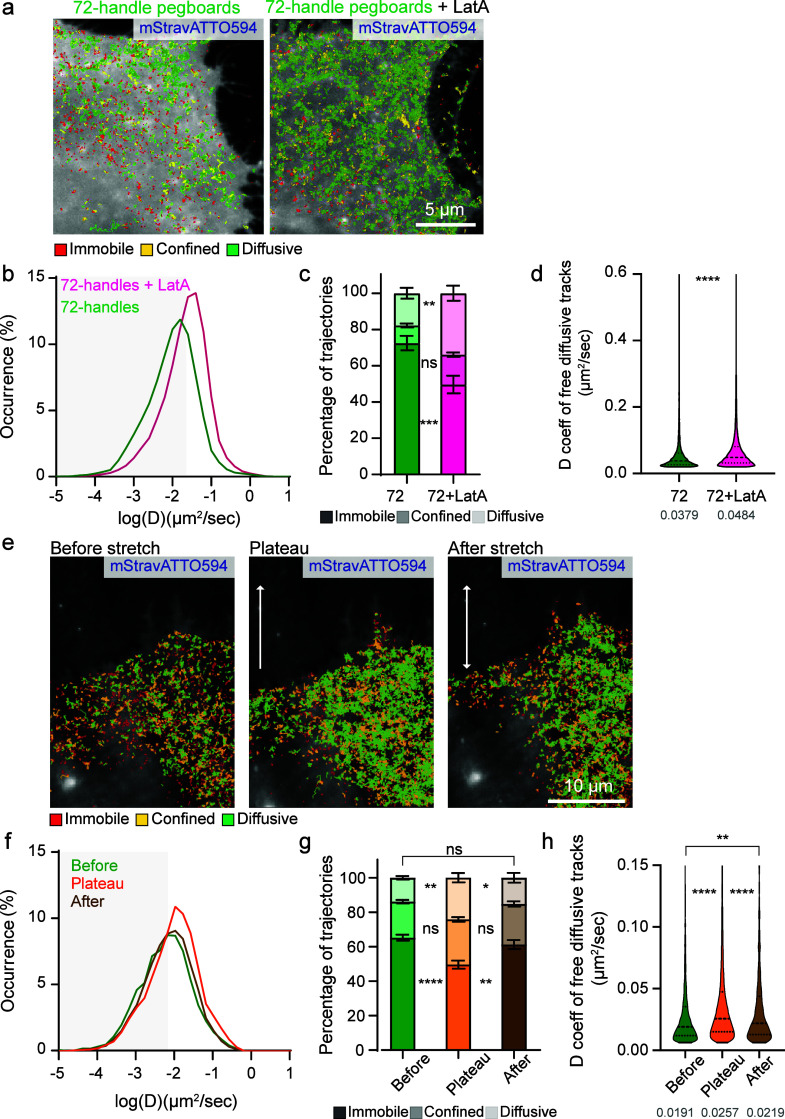
**Membrane nanodomain organization is sensitive to actin and mechanical stimulation. a)** Trajectories of 72-handle DNA origami diffusing on the cell membrane in the presence or absence of LatrunculinA (LatA) overlaid on 6-FAM cell plasma membrane (gray). Trajectories are color coded to show their diffusion modes: diffusive (green), confined (yellow) and immobile (red). **b)** Distributions of the diffusion coefficient D computed from the trajectories of 72-handle DNA origami with or without LatA. **c)** Fractions of tracked 72-handle DNA origami undergoing free diffusion, confined diffusion or immobilization in the plasma membrane with or without LatA actin destabilization. **d)** Diffusion coefficient D for all free diffusive tracks of 72-handles DNA origami in LatA presence or absence. **e)** Representative images of the 6-FAM positive cells superimposed with 72-handles DNA origami tracks before stretch, at the plateau and after stretch. Arrows indicate the stretching and relaxation direction. **f)** Distributions of the diffusion coefficient D computed from the trajectories of 72-handles DNA origami in all the conditions. **g)** Fractions of tracked 72-handles DNA origami undergoing free diffusion, confined diffusion or immobilization in the plasma membrane before stretch, at the plateau and after stretch. **h)** Diffusion coefficient D for all free diffusive tracks of the tested conditions. Data information: mean ± SEM (c,g), median and interquartile range (d,h). Statistics: *n* = 6 (72-handles), *n* = 8 (72-handles + LatA), *n* = 6 (Before), *n* = 4 (Plateau), *n* = 4 (After); c,g) 2-way-ANOVA with Sidak multiple comparisons test, d) number of diffusive tracks 3789 (72-handles), 54126 (72-handles + LatA), Mann–Whitney test, h) number of diffusive tracks 4272 (Before), 6870 (Plateau), 2233 (After), 1-way-ANOVA with Holm-Sidak multiple comparisons test. Abbreviations: LatA, LatrunculinA actin destabilizer.

## Mechano-Stimulation Transiently Regulates Membrane Nanodomain Organization

Building on these observations of dynamic, multifactor confinement, we next investigated how external mechanical forces influence the PM landscape and DNA origami diffusion. Mechanical forces exerted on cells regulate membrane tension,^53^ impact the underlying actin cytoskeleton,^42,50^ and control membrane nanotopography by flattening caveolae or reshaping nanoscale membrane invaginations and evaginations.^54,55^ Moreover, actomyosin-generated forces influence PM nanodomains, controlling their formation and stabilization.^11,18,50^ To test how mechanical force regulates PM nanodomains, we combined cell stretching with DNA origami tracking using a stretching device we recently developed that is compatible with super-resolution microscopy and single-molecule tracking.^42,56^ We tracked 72-handle pegboards on lipid-decorated MEF cells before, during, and after 10% uniaxial stretch in 30 s. Stretching significantly increased the fraction of freely diffusive trajectories with concomitant decreased immobilization at peak stretch compared to unstretched conditions. Importantly, upon relaxation, the diffusive pegboard fraction was restored to prestretch levels (Figure 4e-g). Analysis of diffusion coefficients revealed higher speeds during stretching than before and after relaxation (Figure 4h), while no differences were observed in fold anisotropy for free diffusive and confined tracks (). These results suggest that mechanical force can control PM nanodomain organization by decreasing nanodomain number or density. The rapid and reversible effects indicate an acute membrane or cytoskeletal mechanism rather than the effects due to slower processes such as cholesterol depletion.^2,57^

## Conclusions

This study shows that immobilization and diffusion of our DNA origami pegboards on the PM are influenced by several factors: number and spatial arrangement of lipids on the DNA origami, actin destabilization, and mechanical forces. These findings establish DNA origami pegboards as a robust tool for probing the nanoscale dynamic organization of the PM in living cells. SPT coupled with DNA origami has been used to study enzyme dynamics,^58^ membrane proteins^59^ and to mimic biological membrane components,^60^ while lipophilically anchored DNA nanostructures have been used to act on model^34–37^ and cell membranes.^39,40^ Here, we report the first use of DNA origami to directly interrogate PM nanodomain organization and dynamics. Our approach reveals a PM landscape composed of nanodomains spanning a wide size range, with high density of small nanodomains (5–20 nm), whose organization is partially actin-dependent and reversibly remodeled by mechanical stretch.

By systematically varying the number and spatial distribution of lipid anchors on the DNA origami pegboards, we probed nanodomain size and density at length scales below the optical diffraction limit. The 72-handle design (38.5 nm × 36 nm) exhibited the highest level of immobilization and confinement, consistent with entrapment within either large (>38.5 nm) nanodomains or multiple closely spaced smaller domains. Importantly, if PM nanodomains were predominantly much larger than 38.5 nm, all 8-handle designs would be expected to display similar diffusion behavior, as all anchors would reside within the same domain (Figure 2a, ). Instead, we observed strong configuration-dependent effects: the “packed” 8-handle pegboard (3.5 nm × 21.6 nm) was less immobilized and confined than the “spread” 8-handle design, consistent with membrane organization at length scales below 20 nm. In this scenario, “packed” pegboards can avoid simultaneous engagement with multiple nanodomains, whereas the “spread″ 8-handle and 72-handle designs can effectively be trapped. Consistently, “packed” and “patchy” designs shared similar diffusion and anisotropy, whereas 8-handle “spread” showed enhanced confinement, reduced diffusion, and higher immobilization.

Subtrack analysis further supports a model of dense nanoscale compartmentalization: more time is spent in confined or immobile states than in free diffusion, and 33% of confinement radii fall below the system’s localization precision (<33 nm), with 97% under 100 nm (Figure 3). Our results align with previous reports of nanodomains ranging from 5–30 nm,^18^ or ∼ 20 nm,^19^ as inferred from homo-FRET, STED-FCS, and tracking of GPI-anchored proteins. Larger nanodomains in the 50–100 nm range have also been observed by super-resolution imaging of GPI-anchored proteins using STORM,^13^ NSOM,^10^ and PALM.^61^ However, unlike approaches targeting specific components, our method probes the entire membrane beneath the DNA origami, revealing the density and distribution of small (<20 nm) nanodomains, both abundant and closely spaced.

This range in DNA origami diffusive behavior suggests that observed immobilizations and confinements reflect nanodomain presence rather than extracellular membrane component interactions.^8,12,15^ An alternative or complementary hypothesis is that membrane nanotopographies may also influence DNA origami pegboard diffusion. Given their size and geometry, the DNA origami constructs are unlikely to induce significant membrane curvature or nanotopographical features themselves, as shown for single origami of similar dimensions on model membrane.^35^ Instead, they could act as passive sensors of native membrane nanotopography. Indeed, simulations show that increased membrane curvature reduces the diffusion of membrane-associated proteins^62^ and molecular-dynamics simulations demonstrate that membrane proteins locally reshape the lipid environment creating thickness and curvature gradients.^46^ Beyond nanotopographies, the PM is compartmentalized into 50–200 nm domains created by interactions between the underlying actin cytoskeleton (fence) and transmembrane proteins (pickets).^23,63^ This fence-and-picket organization causes anomalous diffusion of phospholipids and transmembrane proteins over long spatiotemporal scales, whereas free diffusion occurs within individual compartments and hop diffusion enables transitions between adjacent corrals.^23,64^ The observed differences between 8-handle ″packed″ and ″patchy″ versus 8-handle ″spread″ and 72-handle designs may reflect varying probabilities of hopping between actin-defined compartments. Larger or more extensively anchored DNA origami structures likely experience reduced intercompartmental mobility, particularly if corral sizes approach the membrane footprint of the pegboards. We observed changes in rates of DNA origami diffusion and immobilization following both actin destabilization and cell stretching. These findings support the hypothesis that DNA origami diffusive behavior could be regulated by incorporation into lipid nanodomains, interactions with PM nanotopography (e.g., membrane curvature), or the corralling effect, as all these processes are influenced by the actin cytoskeleton and mechanical stimuli.^11,50,54^ ^,^ ^55,65^

Acute actin depolymerization using LatrunculinA^43^ reduced, but did not eliminate, DNA origami-lipid immobilizations and confinements, indicating the coexistence of actin-dependent and actin-independent PM nanodomains. Actin-dependent nanodomains may arise from direct PM-actin coupling,^8,11,12^ ^,^ ^14,15,50^ for example, via linker proteins such as ezrin/radixin/moesin^14,50^ whereas actin-independent nanodomains may be driven by PM nanotopographies,^46^ lipid compositions,^2^ or transmembrane proteins lacking cytoskeletal anchoring.^22^ Because our approach does not rely on tracking individual lipids or proteins, it captures both classes of nanodomains within the same measurement framework.

Using a cell-stretching device we developed,^42,56^ we show that PM nanodomain organization is dynamically and reversibly regulated by mechanical forces. Stretching resulted in a 10% decrease in immobile DNA origami tracks and a significant increase in free diffusion. This could reflect transient nanodomain disassembly due to disconnection from the actin cytoskeleton,^12,50,66^ increased membrane tension,^53,67,68^ altering PM nanotopography^55,69^ or remodeling of cortical actin corralling effect.^65^ PM tension causes caveolae flattening^54,55^ and membrane nanotopography reshaping,^69,70^ and both events may contribute to increased DNA origami mobility. Importantly, diffusion changes were reversible upon relaxation (Figure 4e-h), consistent with transient PM deformations reported in mechanically stressed myoblasts^54^ and fibroblasts.^69,70^ Mechano-induced lipid raft disruption,^13^ caveolae disassembly,^71,72^ and PM nanoscale reshaping,^69^ release PM-associated proteins (e.g., EHD2, Cav1 or ERM proteins)^2,53,71,72^ or recruit PM-bound proteins (e.g., the IRSp53 BAR protein),^69^ potentially triggering mechanotransduction.^72^ Our findings show that mechanical force can rapidly reshape PM nanodomains, potentially modulating membrane-associated signaling.

In summary, our work introduces DNA origami as a new class of nanoscale probes for living-cell plasma membranes. Our data support a PM landscape model characterized by the coexistence of nanodomains larger than 20 nm and a high density of tiny nanodomains (5–20 nm) (Figure S5). These nanodomains are partially stabilized by the underlying actin cytoskeleton, and mechanical forces can transiently remodel them. By enabling controlled nanoscale interrogation of the plasma membrane in living cells, this approach provides access to membrane organization that is inaccessible to existing techniques and reveals key features of PM nanodomain dynamics. This work establishes a foundation for more sophisticated DNA origami-based PM probes. Recent advances in the field produced a rapidly growing library of origami particles with larger surface areas and more complex topographies.^27,28^ These designs could be used in future studies to survey larger PM areas or to induce local membrane curvature.

## Supplementary Material















## Notes

Detailed experimental methods; Figure S1–S2 (diffusive behavior of 1C and 2C lipid anchors in the presence and absence of 12-handle DNA origami pegboards); Figure S3–S4 (DNA origami anisotropy as a function of handle distribution and mechanical perturbation of the cell); Figure S5–S6 (graphical representation of the proposed model and Cadnano design files) (PDF)
Movie S1 (2C lipid-anchor labeled with Cy3b Imager) (AVI)
Movie S2 (DNA origami pegboards labeled with mStravATTO594: S2, 8-handle packed) (AVI)
Movie S3 (DNA origami pegboards labeled with mStravATTO594: S3, 8-handle spread) (AVI)
Movie S4 (DNA origami pegboards labeled with mStravATTO594: S4, 72-handle spread) (AVI)
Movies S5 (DNA origami pegboards labeled with mStravATTO594: S5, 72-handle spread with subtrack analysis) (AVI)
Table S1 (DNA origami pegboard fabrication sequences) (XLSX)
