# Supplementary material for: Using DNA Origami to Study Nanoscale Organization of Plasma Membranes
Source: Nano Lett. 2026 Apr 20;26(25):8064–74. doi: 10.1021/acs.nanolett.6c00255 (PMC13329991; doi:10.1021/acs.nanolett.6c00255)
Supplement: Supplementary file 1 [file nl6c00255_si_001.pdf]

SUPPORTING INFORMATION

Using DNA origami to study nanoscale organization of plasma membranes

Eloina Corradi[1,†,*], Konlin Shen[2,†], Zeynep Karatas[1], Maureen Cercy[1], Thomas Schlichthaerle[3,4], Margaux Caumont[1], Melissande Osouf[1], Brune Vialet[5], Philippe Barthelemy[5], Morgane Rosendale[1], Adiyodi Veetil Radhakrishnan[1,6], Tianchi Chen[1], Ralf Jungmann[3,4], Arnaud Gissot[5], Shawn M. Douglas[2,‡], Grégory Giannone[1,‡,*].

† These authors contributed equally to this work (co–first authors).
‡ These authors jointly supervised this work (co–senior authors).
* To whom correspondence may be addressed. Email: gregory.giannone@u-bordeaux.fr and eloina.corradi@u-bordeaux.fr

[1] University Bordeaux, CNRS, IINS, UMR 5297, Bordeaux, F-33000, France
[2] Dept. of Cellular and Molecular Pharmacology, University of California, San Francisco, San Francisco, CA, 94158, USA
[3] Faculty of Physics and Center for Nanoscience, LMU Munich, Munich, 80539, Germany
[4] Research Group Molecular Imaging and Bionanotechnology, Max Planck Institute of Biochemistry, Martinsried, 82152, Germany
[5] ARNA, INSERM U1212, CNRS 5320, Université de Bordeaux, Bordeaux, F-33076, France
[6] Somaiya Centre for Integrated Science Education and Research (SciSER®), Somaiya Vidyavihar University, Mumbai, Maharashtra, Bharat, 400077, India

Materials and methods

Cell culture Immortalized Mouse Embryonic Fibroblasts (MEF) were cultured in DMEM (Gibco) with 10% v/v Fetal Bovine Serum (FBS). On the day of the experiments, the cells were detached from the extracellular matrix using Trypsin/EDTA 0.05% for 2 min (Gibco). The trypsin was inactivated using soybean trypsin inhibitor (STI, Sigma) or with 10% (v/v) FBS DMEM. When trypsin was inactivated with 10% (v/v) FBS DMEM, cells were washed twice by consecutive cycles of centrifugation, supernatant removal, and resuspension in Ringer serum-free medium (150 mM NaCl, 5 mM KCl, 2 mM $CaCl_2$, 2 mM $MgCl_2$, 10 mM HEPES, 11 mM Glucose, pH 7.4) to avoid any trace of serum. 50k cells were seeded on human fibronectin (10 µg/ml, Sigma) coated 18 mm 1.5 H coverslips and kept in Ringer medium at 37°. Experiments were performed 3-5 hours after cell plating.
When treated with Latrunculin A (LatA) prior to live imaging cells were incubated for 5-10 minute with 1 µM LatA (Tocris).

DNA origami DNA origami pegboards were fabricated as described in previous works[1,2]. Briefly, DNA origami pegboards were designed using Cadnano (Fig.S6)[3]. The pegboards were assembled from p8064 DNA origami scaffold (Tilibit), and chemically synthesized staple DNA

oligonucleotides (Integrated DNA Technologies/IDT). Staples and scaffold were mixed at a molar ratio of 10:1 and folded in 12 mM Mg DNA origami folding buffer (12 mM Mg, 5 mM Tris, 1 mM EDTA). To fold the origami, the following steps were performed on a thermocycler:

1. hold at 65°C for 15'
2. drop to 60°C and hold for 1 hr
3. decrease temperature by 1°C and hold for 1 hr (repeat this step 19 more times, with a final hold temperature of 40°C)
4. hold at 25°C.

Folded origami were purified by PEG fractionation, then validated using agarose gel electrophoresis and negative stain transmission electron microscopy (TEM). All sequences used in this work can be found in the supplement (Table S1).

Lipid anchor Oligonucleotide synthesis was performed on an H8 automated synthesizer (K&A Labs, Germany) using the conventional phosphoramidite methodology on a one µmole scale. Trichloroacetic acid (TCA, Glen Research, USA) (3% in dichloromethane) was used for detritylation, 0.25 M 5-Benzylthio-1H-tetrazole (BTT, Glen Research) in dry acetonitrile was used as an activator, and oxidation was achieved using 0.02 M iodine in tetrahydrofuran/water/pyridine (Glen Research). The capping was achieved using a mixture of an acetic anhydride solution in THF (Cap A, Glen Research) and 10% 1-methylimidazole in THF/pyridine (Cap B, Glen Research). Pre-packed nucleoside 1000Å CPG (LINK, Scotland) and fast deprotecting β-cyanoethyl phosphoramidite monomers (Bz-dA, Ac-dC, dmf-dG, dT, Glen Research) were used to synthesize DNA. The monomers were dissolved in anhydrous MeCN (0.067 M) immediately prior to use. The fluorescein (6-FAM) was directly bound to the CPG solid support (3'-Fluorescein CPG), and the hexaethylene glycol (HEG) spacer was incorporated to isolate the origami docking oligonucleotide sequence from the lipidic segment of the lipid-DNA conjugate. All non-lipidic phosphoramidites and 6-FAM solid supports were purchased from (LINK).
Lipidic modifications of the oligonucleotide sequences were incorporated during the last cycle of the automated DNA synthesis with 2 previously reported custom-made phosphoramidites: one with a simple C18 saturated alkyl modification and the second one with a ketal modified uridine derivative [4].

The lipid-oligonucleotide sequences synthesized were: 5'-(C18)-TTTCTTCATTA–HEG-TTCCTCTACCACCTACATCACTT-(6 FAM)-3'; 5'-(ketal)-TTTCTTCATTA–HEG-TTCCTC TACCACCTACATCACTT-(6 FAM)-3' used in combination with the 12-handles origami (Fig.S2), and 5' (ketal)-TTTCTTCATTA–HEG-AAGATGAGGTAGATGGTT-(6 FAM)-3' with all the other origami designs (Fig.1-4, Fig.S3-6). Cleavage and deprotection were performed according to supplier protocol. The crude oligonucleotide solution was concentrated under reduced pressure and redissolved in water (0.5 mL). The oligonucleotide sequences were finally dialyzed first against 50 mM NaCl and then twice against water. The oligonucleotide concentrations were determined from the absorbance value at 260 nm and the oligonucleotide's epsilon (molar extinction coefficient). These values were calculated using the Integrated DNA Technology online oligo analyzer tool which uses the standard nearest neighbor method.

Single lipid and DNA origami tracking For single particle tracking with DNA-PAINT (DNA-PAINT-SPT), cells were incubated with lipid anchors (2 µM) diluted in Ringer medium (for single lipids) or in 10 mM MgCl2 Ringer medium (for DNA origami) at room temperature for 15 minutes and washed 3 times with the same dilution medium to remove free lipids not inserted in the plasma membrane. For single lipid tracking experiments (Fig.1, Fig.S1) 0.5-1 nM cy3b-Imager P3 (Massive Photonics) was added to the cells and acquired at room temperature without further incubation. For single-particle tracking of DNA origami (Fig.1-4), 2 nM biotinylated DNA origami was mixed with 1 µM mStravATTO594 for imaging. The mix, diluted in 10 mM MgCl2 Ringer medium, was added to the cell with lipid anchors. DNA origami and mStravATTO594 were used at a final concentration during acquisition of 1 nM and 0.5 µM, respectively. Cells were imaged at room temperature in an open chamber (Ludin chamber, Life Imaging Services, Switzerland) mounted on an inverted motorized microscope Nikon Ti equipped with a 100x 1.45 NA PL-APO objective, an automatic perfect focus system, and a Total Internal Reflection Fluorescence (TIRF) illumination module. The angle of the incident laser beam was adjusted to selectively illuminate the surface of the plasma membrane at the periphery of the cell, where the sample thickness allows a proper TIRF illumination. Cy3b imager P3 (for single lipid tracking) or mStravATTO594 (for DNA origami) were excited at 561 nm (5 mW at the objective), keeping the single molecule regime during multiple frames. The fluorescence was collected by the combination of a dichroic mirror and emission filters (D101-R561 and F39-61,7 respectively, Chroma, USA) and a sensitive EMCCD (Evolve, Photometric, USA). 5 streams of 4000 frames each were acquired. Fluorescein (6-FAM) (on the lipid anchor) was excited using a 488 nm laser. The acquisition was driven by Metamorph software (Molecular Device, USA) in streaming mode at 50 Hz, 20 ms exposure time (for the single lipids and DNA origami, Fig.S1d-f, Fig.1-4, Figs.S3 and S4) or 500 Hz, 2 ms exposure time (single lipids control experiment, Fig.S1h-j).

DNA origami tracking on stretching devices Stretching devices were built as previously[5,6] . MEF cells culture and DNA origami labelling was done as for coverglass. The strain of the uniaxial stretch was calibrated using fluorescence beads (Tetraspeck, ThermoFisher) seeded on the PDMS substrate. As before, cells were imaged at room temperature on an inverted motorized microscope Nikon Ti equipped with a 100x 1.45 NA PL-APO objective, an automatic perfect focus system, and TIRF illumination module. mStravATTO594 were excited at 561 nm (12 mW at the objective) keeping the single molecule regime during multiple frames. Fluorescence data were collected using a combination of a dichroic mirror and emission filters (mEOS filter, Semrock FF02-617/73-25) and a sensitive a sensitive scientific complementary metal-oxide semiconductor sCMOS (ORCA-Flash4.0, Hammamatsu). Acquisition was driven by Metamorph software (Molecular Device, USA) in streaming mode. 2 streams of 4000 frames were acquired with a 50 ms exposure time for each step of the stretching protocol: 2 streams before, 2 streams at the stretching plateau and 2 streams at the relaxation. The uniaxial mechanical stretch applied was a single 10% stretch performed in 30 sec.

Tracking analysis Single molecule localization and tracking was performed using PALMTracer software, a MetaMorph (Molecular Devices, Sunnyvale, USA) add-on developed at the Interdisciplinary Institute of Neuroscience[7]. For stretching experiments, beads were used to register the localizations using the registration option within PALMTracer. Under the experimental settings described above, and the basal camera noise measured experimentally, the resolution of the whole system on coverglass (microscope equipped with a EMCCD (Evolve) camera, pixel size of 160 nm) was 79.6 nm and on the stretching devices (microscope equipped with a sCMOS (ORCA-Flash4.0, Hammamatsu) camera, pixel size of 130 nm) was 71.2 nm. The resolution was determined to be 2.355 $\boldsymbol{\sigma}_{xy}$, where $\boldsymbol{\sigma}_{xy}$ is the pointing accuracy of the Gaussian fitting estimated with Thunderstorm [8]. Therefore, the inference of nanodomains smaller than the resolution of the system does not rely on the optical localization precision but instead derives from geometry-dependent interactions between DNA origami platforms with defined footprints and the membrane landscape. The trajectories were analyzed specifically inside the cell, thus removing the contribution of immobile detections on the coverglass, by using as a reference the segmented GFP image (derived from the alexa-488 of the lipid anchors). We analyzed trajectories lasting at least 10 points (i.e. 200 ms, for all experiments; 500 ms, for the stretching experiment) with a custom Matlab code, as previously done[8,9]. The Matlab algorithm computes the mean square displacement (MSD) of the particles and the corresponding diffusion coefficient defined as the slope of the affine regression line of the MSD fitted for the four first values over time[8]. We use step angle anisotropy to analyze the confinement of trajectories[9,10]. First, step angles between consecutive steps of only diffusive trajectories and steps greater than the resolution limit are collected from each set of experiments. The distribution of the angles is drawn as a rose plot. Anisotropy was quantified from the distribution of step angles by calculating the ratio of steps oriented around 180° to those around 0°. Angles between 150–210° were classified as 180°-aligned steps, while angles between 330–360° and 0–30° were classified as 0°-aligned steps. For sub-trajectory analysis, we only analyzed tracks fulfilling these conditions: tracks longer than 50 frames, presenting local confinement (i.e. region of immobilization and confined diffusion) interspersed with free diffusive regions. For the main MSD analysis, a minimal length of 10 frames was required for each region of local confinement to be included in the analyses. Finally, the spatial extent of confinement was estimated by calculating the radius of confinement (R_{conf}) as square root of the MSD plateau value as previously described[8,9].

Statistical analysis All data were analyzed with Prism (GraphPad 7). Respective n values, data representation (i.e. median, mean, errors) and statistical test used are specified in the Figure legends. For all tests, the significance level was $\alpha = 0.05$. Resulting p-values are indicated as follows n.s when $p > 0.05$; * when $0.01<P<0.05$; ** when $0.001<P<0.01$; *** when $P<0.0001$.

Reference

1. Dong, R. *et al.* DNA origami patterning of synthetic T cell receptors reveals spatial control of the sensitivity and kinetics of signal activation. *Proc. Natl. Acad. Sci.* **118**, e2109057118 (2021).
2. Niekamp, S., Stuurman, N. & Vale, R. D. A 6-nm ultra-photostable DNA FluoroCube for fluorescence imaging. *Nat. Methods* **17**, 437–441 (2020).
3. Douglas, S. M. *et al.* Rapid prototyping of 3D DNA-origami shapes with caDNAno. *Nucleic Acids Res.* **37**, 5001–5006 (2009).
4. Vialet, B., Gissot, A., Delzor, R. & Barthélémy, P. Controlling G-quadruplex formation via lipid modification of oligonucleotide sequences. *Chem. Commun.* **53**, 11560–11563 (2017).
5. Massou, S. *et al.* Cell stretching is amplified by active actin remodelling to deform and recruit proteins in mechanosensitive structures. *Nat. Cell Biol.* **22**, 1011–1023 (2020).
6. Nunes Vicente, F. *et al.* A micromechanical cell stretching device compatible with super-resolution microscopy and single protein tracking. *Researchsquare* (2020) doi:10.21203/rs.3.pex-961/v1.
7. Butler, C. *et al.* Multi-Dimensional Spectral Single Molecule Localization Microscopy. *Front. Bioinforma.* **2**, 813494 (2022).
8. Rossier, O. *et al.* Integrins β 1 and β 3 exhibit distinct dynamic nanoscale organizations inside focal adhesions. *Nat. Cell Biol.* **14**, 1057–1067 (2012).
9. Chen, T. *et al.* Actin-driven nanotopography promotes stable integrin adhesion formation in developing tissue. *Nat. Commun.* **15**, 8691 (2024).
10. Hansen, A. S., Amitai, A., Cattoglio, C., Tjian, R. & Darzacq, X. Guided nuclear exploration increases CTCF target search efficiency. *Nat. Chem. Biol.* **16**, 257–266 (2020).

Supplementary Movies

Movie S1 – Lipid anchors with two aliphatic chains (2C) imaged by Cy3b imager complementarity through SPT-DNA-PAINT. Raw data of the cell showed in Fig.1d, left.

Movie S2 – Imaging of "packed" 8-handle DNA origami pegboards labelled with mStravATTO594. Raw data of the cell showed in Fig.2c.

Movie S3 – Imaging of "spread" 8-handle DNA origami pegboards labelled with mStravATTO594. Raw data of the cell showed in Fig.2c.

Movie S4 – Imaging of "packed" 72-handle DNA origami pegboards labelled with mStravATTO594. Raw data of the cell showed in Fig.2c.

Movie S5 – Sub-track diffusion coefficient as a function of time and space for a representative trajectory. Trajectory showed in Fig.3a.

Supplementary Table

Table S1 – Table reporting all sequences for DNA origami pegboards fabrication.

Supplementary Figures

Figure S1

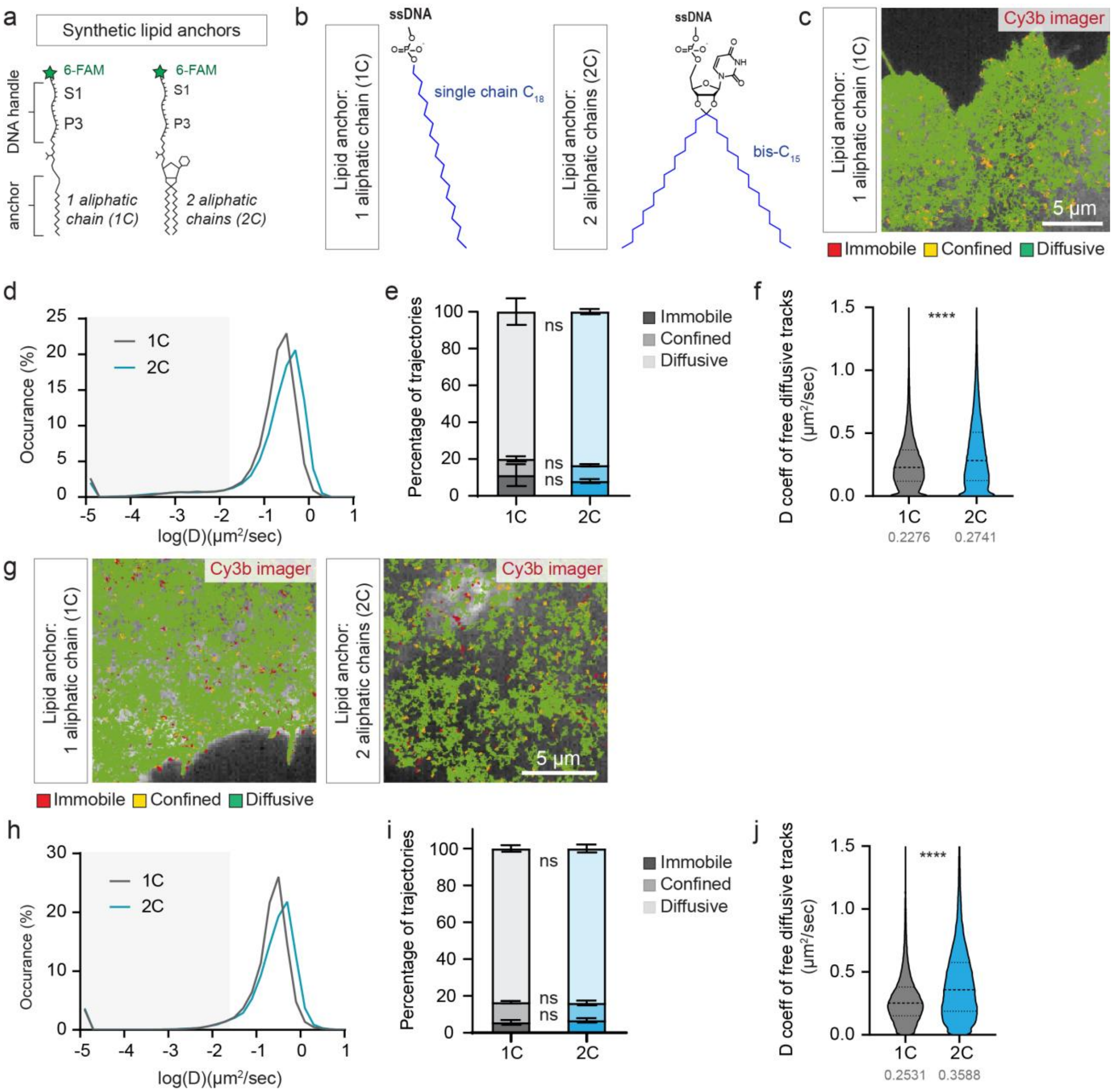

Figure S1. Lipid anchors freely diffuse on the plasma membrane. a,b) Graphical representation of lipid anchor structures (ssDNA sequence are reported in the method section). **c-f)** DNA-PAINT tracking of 1C and 2C lipid anchors acquired at 50Hz in MEFs. c) Trajectories of single lipid anchor 1C overlaid on 6-FAM cell plasma membrane (gray) – note that trajectories of single lipid anchor 2C are shown in Figure 1d. Trajectories are color coded to show their diffusion modes: diffusive (green), confined (yellow) and immobile (red). d) Distributions of the diffusion coefficient D computed from the trajectories of lipid anchor 1C and 2C. e) Fractions of tracked 1C and 2C lipids undergoing free diffusion, confined diffusion or immobilization in the plasma membrane. f) Diffusion coefficient D for all free diffusive tracks of 1C and 2C lipids. Data for 2C lipid anchors (d-f) are reported in Figure 1 for direct comparison in the presence/absence of DNA origami. **g-j)** DNA-PAINT-SPT of 1C and 2C lipids acquired at 500 Hz: g) Trajectories of single lipid 1C and 2C overlaid on 6-FAM cell plasma membrane (gray). Trajectories are color coded to show their diffusion modes: diffusive (green), confined (yellow) and immobile (red). h) distributions of the diffusion coefficient

D computed from the trajectories of lipid anchor 1C and 2C, i) fraction of tracked 1C and 2C lipids undergoing free diffusion, confined diffusion or immobilization in the cellular plasma membrane, j) diffusion coefficient D for all free diffusive tracks of 1C and 2C lipids.

Data information: mean ± SEM (e,i), median and interquartile range (f,j). Statistics: d-f) n=5, h-j) n=6, fe,i) 2-way-ANOVA with Sidak multiple comparisons test, f) number of diffusive tracks 120755 (1C), 132543 (2C), unpaired t-test, j) number of diffusive tracks 82764 (1C), 82519 (2C), unpaired t-test. Abbreviations: 1C, lipid anchor with one aliphatic chain; 2C, lipid anchor with two aliphatic chains; cy3b, cyanine 3b.

Figure S2

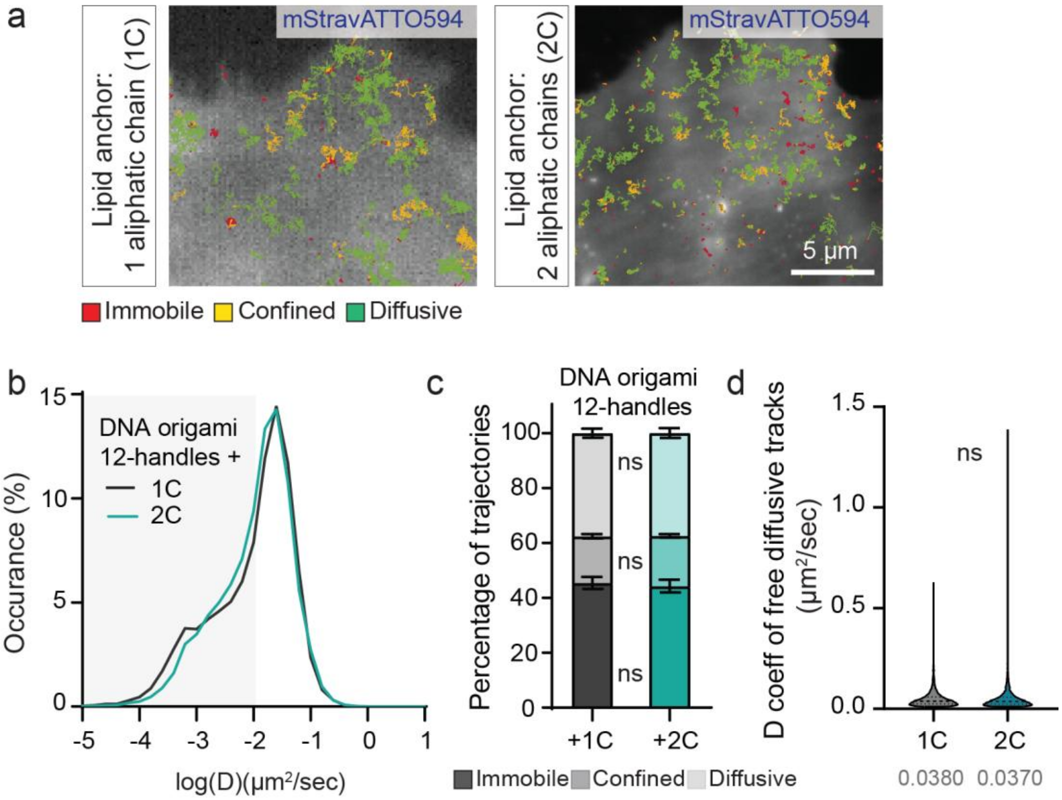

Figure S2. DNA origami can be used as a probe for cell membranes. a) Trajectories of 12-handles DNA origami, using either 1C or 2C lipid anchor, overlaid on 6-FAM cell plasma membrane (gray). Trajectories are color coded to show their diffusion modes: diffusive (green), confined (yellow) and immobile (red). **b)** Distributions of the diffusion coefficient D computed from the trajectories of 12-handles DNA origami using either 1C or 2C lipid anchor. **c)** Fractions of tracked 12-handles DNA origami combined with 1C or 2C lipids anchor undergoing free diffusion, confined diffusion or immobilization in the plasma membrane. **d)** Diffusion coefficient D for all free 12-handles DNA origami diffusive tracks combined with 1C or 2C lipids anchor.

Data information: mean ± SEM (c), median and interquartile range (d). Statistics: b-d) n=23 (1C) and n=18 (2C); c) 2-way-ANOVA with Sidak multiple comparisons test, d) number of diffusive tracks 13953 (1C), 13444 (2C), unpaired t-test. Abbreviations: 1C, lipid anchor with one aliphatic chain; 2C, lipid anchor with two aliphatic chains.

Figure S3

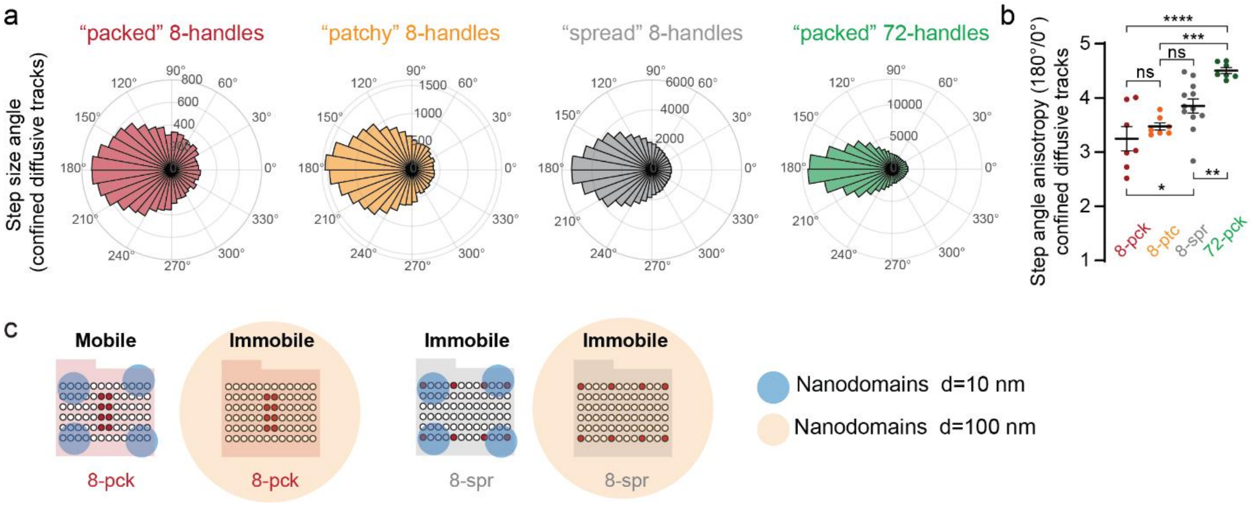

Figure S3. The spatial arrangement of DNA origami handles for lipid anchors influences diffusion and anisotropy. a) Representative step size angle distribution for the confined tracks of the tested DNA origami. **b)** Step angle anisotropy of all the confined tracks. **c)** Visual summary of the results.

Data information: mean ± SEM. Statistics: n=7 (8-pck), n=7 (8-ptc) n=12 (8-spr), n=7 (72-pck), b) ordinary one-way ANOVA Tukey multiple comparisons test, each dot corresponds to the step angle anisotropy per single cell. Abbreviations: pck, packed; ptc, patchy; spr, spread.

Figure S4

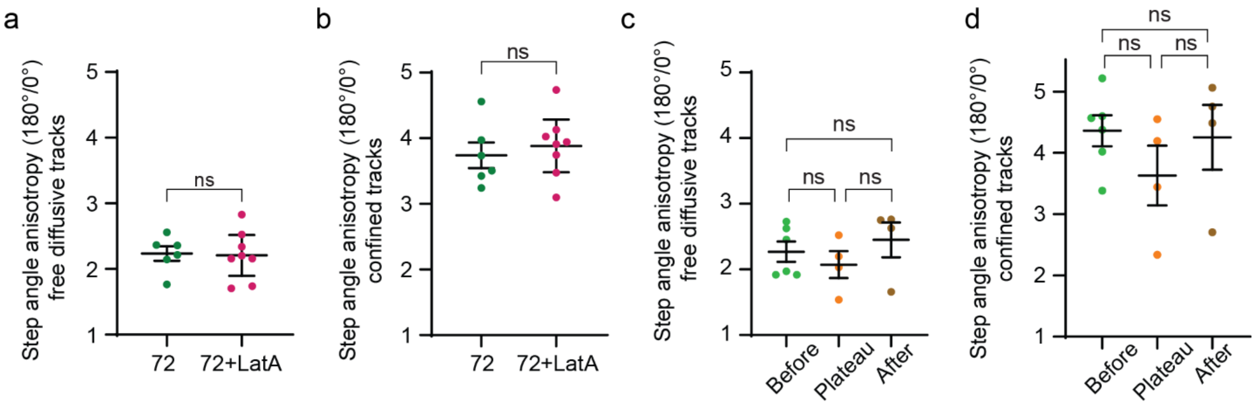

Figure S4. Membrane nanodomains partially rely on actin and are sensitive to mechanical stimulation. a-d) Step angle anisotropy of all the 72-handles DNA origami free diffusive tracks (a,c) and confined tracks (b,d) in the presence (+LatA) or absence of LatrunculinA and (a,b) before stretch, at the plateau and after stretch (c,d).

Data information: mean ± SEM. Statistics: n=6 (no LatA), n=8 (+ LatA), n=6 (Before), n=4 (Plateau), n=4 (After); (a,b) unpaired t-test, (c,d) Kruskal-Wallis test Dunn's multiple comparisons test, each dot corresponds to the step angle anisotropy per single cell. Abbreviations: LatA, LatrunculinA actin destabilizer.

Figure S5

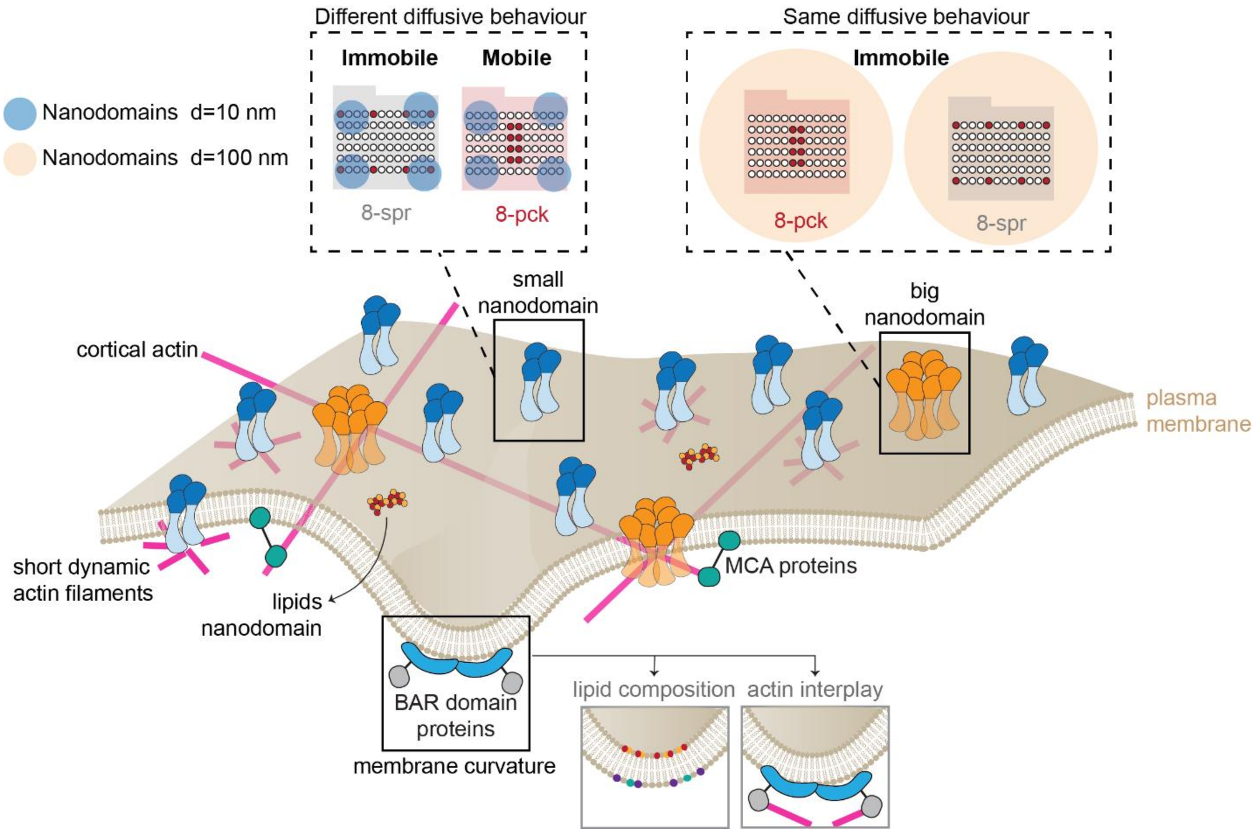

Figure S5. DNA origami to study the nanoscale organization of the plasma membrane. Schematic of the principle of DNA origami probing the plasma membrane nanodomains of different sizes (blue circles, nanodomains of 10 nm in diameter; light orange circles, nanodomains of 100 nm in diameter). DNA origami reveal a plasma membrane characterized by high density of small nanodomains (5-20 nm), dependent and independent on the actin cytoskeleton (magenta).

Figure S6

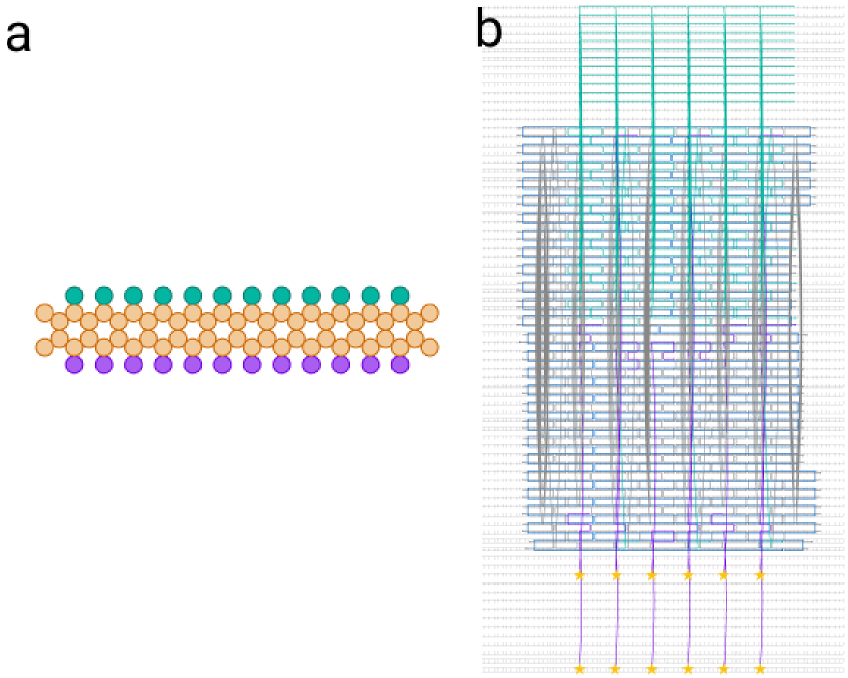

Figure S6. Cadnano files depicting the cross sectional helix view (a) and the staple routing path (b). Teal helices and staples represent staples bearing ssDNA handles for lipid anchors. Purple helices and staples represent biotinylated staples for conjugating mStrav-ATTO594. Biotin sites are denoted by stars and are located at the 5’ ends of the purple staples.
